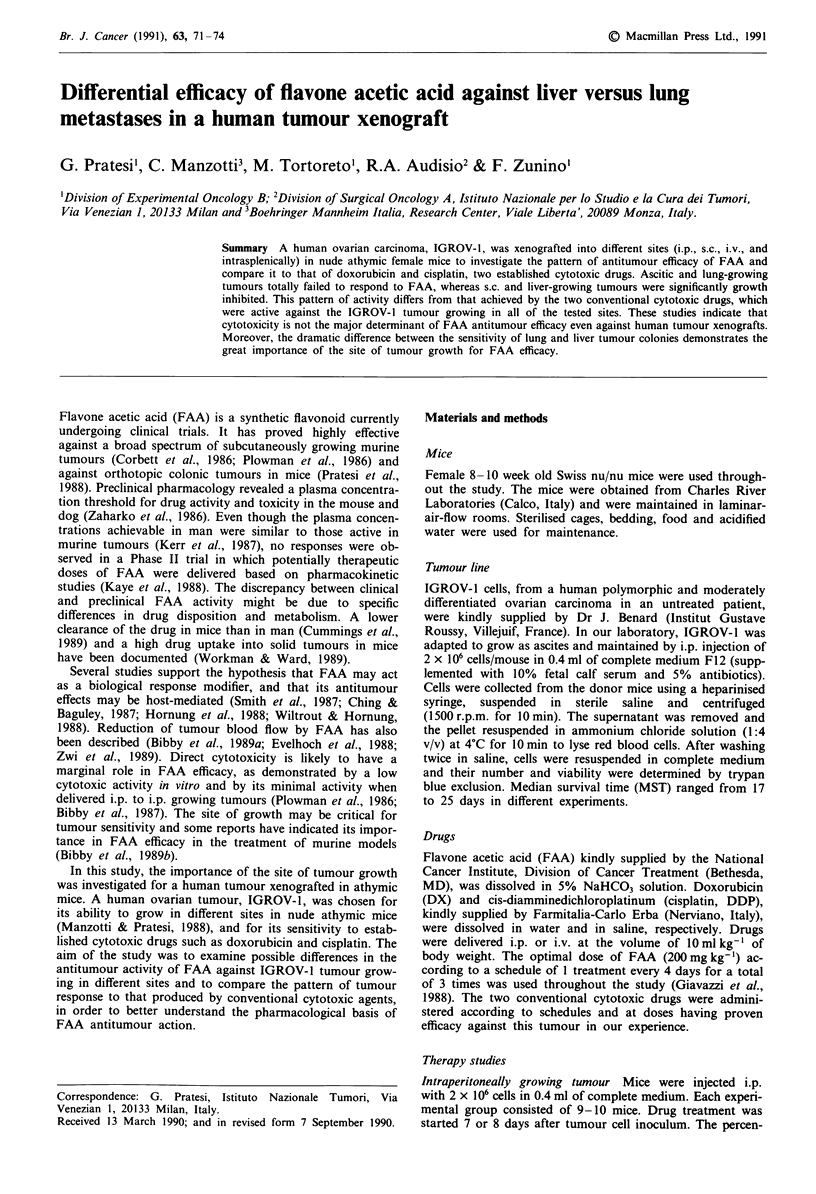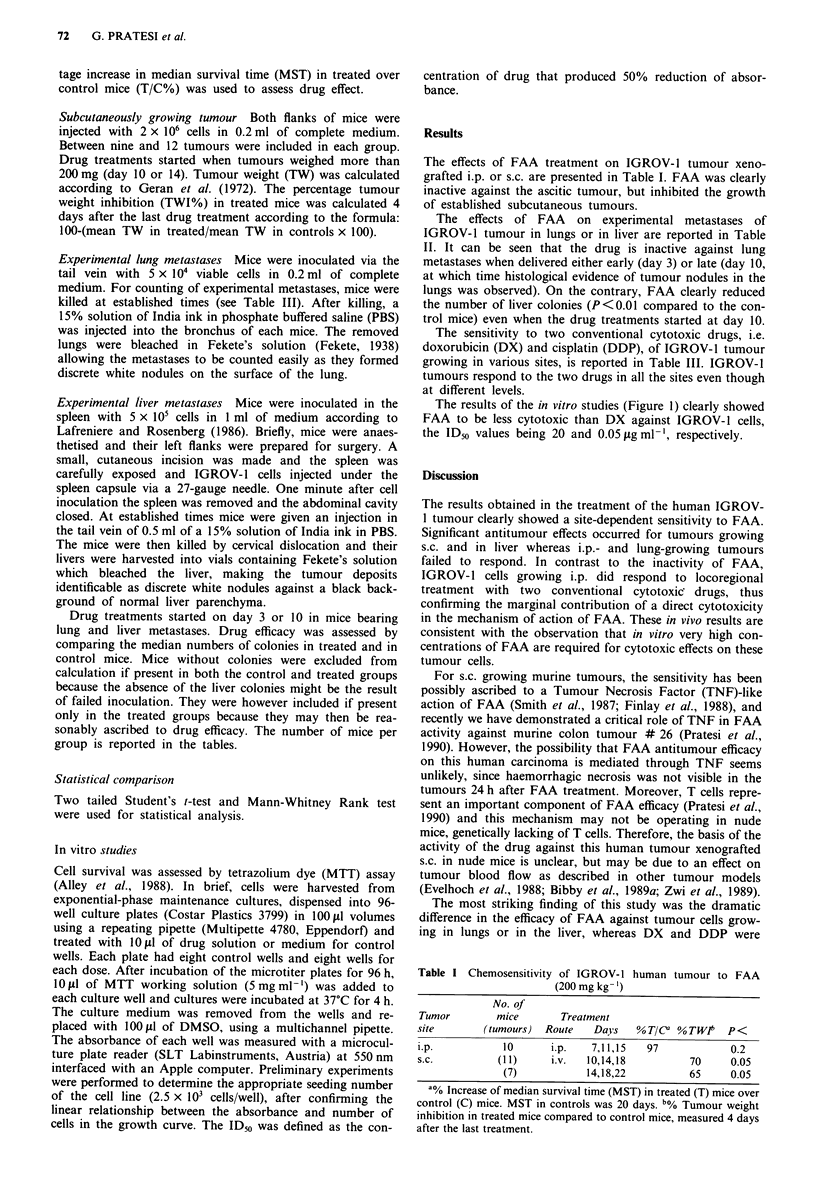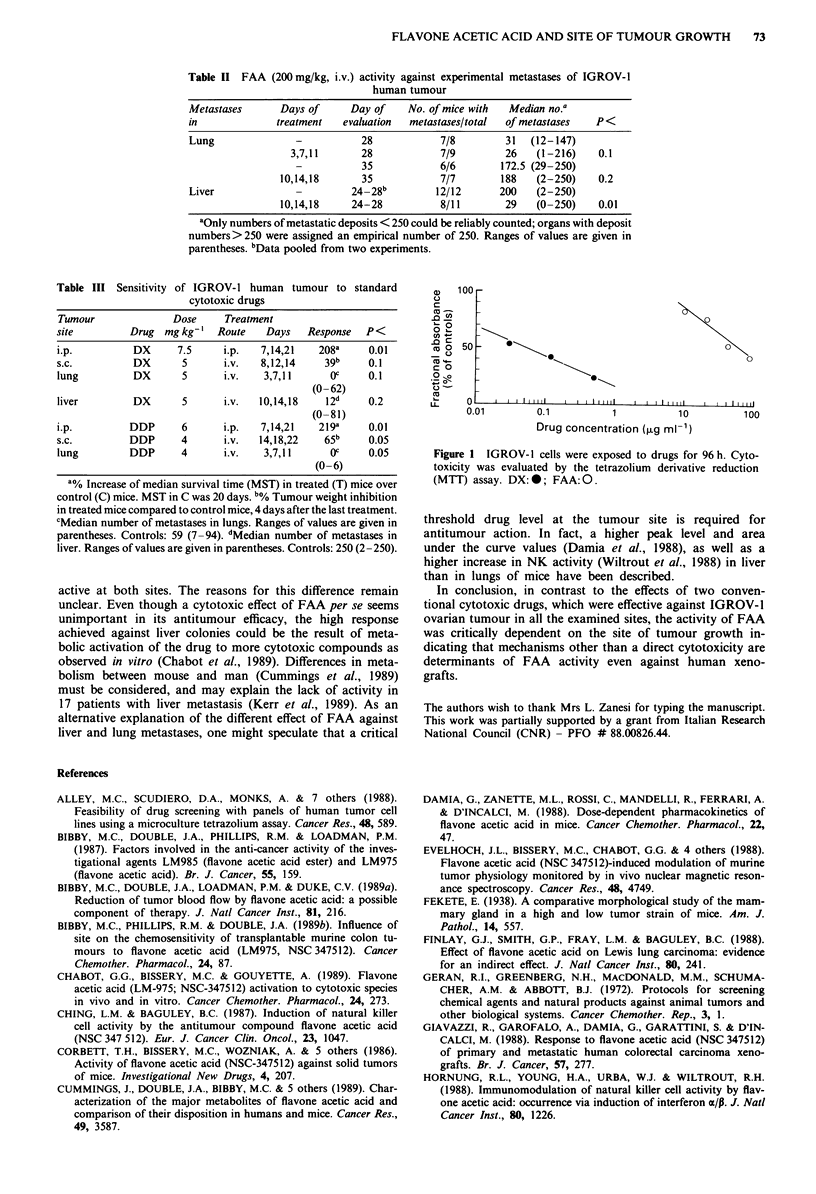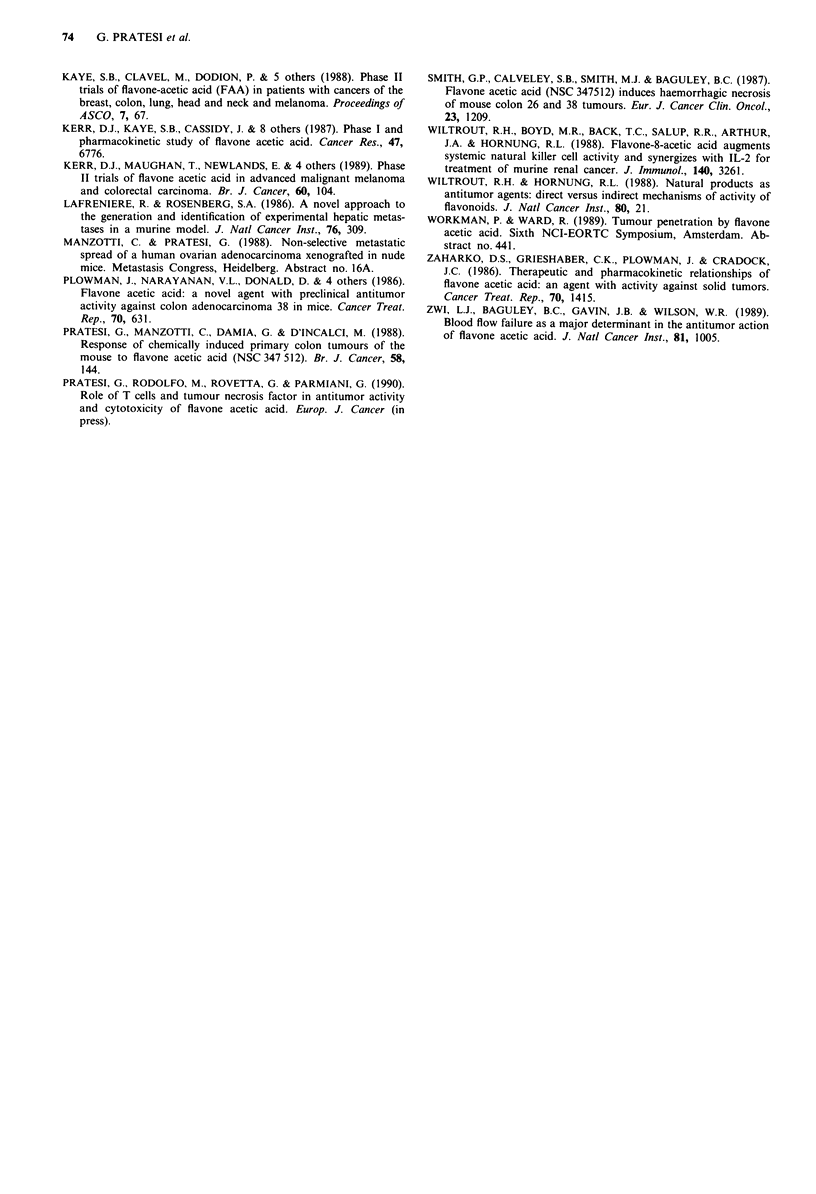# Differential efficacy of flavone acetic against liver versus lung metastases in a human tumour xenograft.

**DOI:** 10.1038/bjc.1991.15

**Published:** 1991-01

**Authors:** G. Pratesi, C. Manzotti, M. Tortoreto, R. A. Audisio, F. Zunino

**Affiliations:** Division of Experimental Oncology B, Istituto Nazionale per lo Studio e la Cura dei Tumori, Milan, Italy.

## Abstract

A human ovarian carcinoma, IGROV-1, was xenografted into different sites (i.p., s.c., i.v., and intrasplenically) in nude athymic female mice to investigate the pattern of antitumour efficacy of FAA and compare it to that of doxorubicin and cisplatin, two established cytotoxic drugs. Ascitic and lung-growing tumours totally failed to respond to FAA, whereas s.c. and liver-growing tumours were significantly growth inhibited. This pattern of activity differs from that achieved by the two conventional cytotoxic drugs, which were active against the IGROV-1 tumour growing in all of the tested sites. These studies indicate that cytotoxicity is not the major determinant of FAA antitumour efficacy even against human tumour xenografts. Moreover, the dramatic difference between the sensitivity of lung and liver tumour colonies demonstrates the great importance of the site of tumour growth for FAA efficacy.


Br. J. Cancer (1991), 63, 71–74 © Macmillan Press Ltd., 1991

### Differential efficacy of flavone acetic acid against liver versus lung metastases in a human tumour xenograft

G. Pratesi[1], C. Manzotti[3], M. Tortoreto[1], R.A. Audisio[2] & F. Zunino[1]

[1]*Division of Experimental Oncology B;* [2]*Division of Surgical Oncology A, Istituto Nazionale per lo Studio e la Cura dei Tumori, Via Venezian 1, 20133 Milan and* [3]*Boehringer Mannheim Italia, Research Center, Viale Liberta', 20089 Monza, Italy.*

**Summary** A human ovarian carcinoma, IGROV-1, was xenografted into different sites (i.p., s.c., i.v., and intrasplenically) in nude athymic female mice to investigate the pattern of antitumour efficacy of FAA and compare it to that of doxorubicin and cisplatin, two established cytotoxic drugs. Ascitic and lung-growing tumours totally failed to respond to FAA, whereas s.c. and liver-growing tumours were significantly growth inhibited. This pattern of activity differs from that achieved by the two conventional cytotoxic drugs, which were active against the IGROV-1 tumour growing in all of the tested sites. These studies indicate that cytotoxicity is not the major determinant of FAA antitumour efficacy even against human tumour xenografts. Moreover, the dramatic difference between the sensitivity of lung and liver tumour colonies demonstrates the great importance of the site of tumour growth for FAA efficacy.

Flavone acetic acid (FAA) is a synthetic flavonoid currently undergoing clinical trials. It has proved highly effective against a broad spectrum of subcutaneously growing murine tumours (Corbett *et al.*, 1986; Plowman *et al.*, 1986) and against orthotopic colonic tumours in mice (Pratesi *et al.*, 1988). Preclinical pharmacology revealed a plasma concentration threshold for drug activity and toxicity in the mouse and dog (Zaharko *et al.*, 1986). Even though the plasma concentrations achievable in man were similar to those active in murine tumours (Kerr *et al.*, 1987), no responses were observed in a Phase II trial in which potentially therapeutic doses of FAA were delivered based on pharmacokinetic studies (Kaye *et al.*, 1988). The discrepancy between clinical and preclinical FAA activity might be due to specific differences in drug disposition and metabolism. A lower clearance of the drug in mice than in man (Cummings *et al.*, 1989) and a high drug uptake into solid tumours in mice have been documented (Workman & Ward, 1989).

Several studies support the hypothesis that FAA may act as a biological response modifier, and that its antitumour effects may be host-mediated (Smith *et al.*, 1987; Ching & Baguley, 1987; Hornung *et al.*, 1988; Wiltrout & Hornung, 1988). Reduction of tumour blood flow by FAA has also been described (Bibby *et al.*, 1989*a*; Evelhoch *et al.*, 1988; Zwi *et al.*, 1989). Direct cytotoxicity is likely to have a marginal role in FAA efficacy, as demonstrated by a low cytotoxic activity *in vitro* and by its minimal activity when delivered i.p. to i.p. growing tumours (Plowman *et al.*, 1986; Bibby *et al.*, 1987). The site of growth may be critical for tumour sensitivity and some reports have indicated its importance in FAA efficacy in the treatment of murine models (Bibby *et al.*, 1989*b*).

In this study, the importance of the site of tumour growth was investigated for a human tumour xenografted in athymic mice. A human ovarian tumour, IGROV-1, was chosen for its ability to grow in different sites in nude athymic mice (Manzotti & Pratesi, 1988), and for its sensitivity to established cytotoxic drugs such as doxorubicin and cisplatin. The aim of the study was to examine possible differences in the antitumour activity of FAA against IGROV-1 tumour growing in different sites and to compare the pattern of tumour response to that produced by conventional cytotoxic agents, in order to better understand the pharmacological basis of FAA antitumour action.

Correspondence: G. Pratesi, Istituto Nazionale Tumori, Via Venezian 1, 20133 Milan, Italy.
Received 13 March 1990; and in revised form 7 September 1990.

### Materials and methods

### Mice

Female 8–10 week old Swiss nu/nu mice were used throughout the study. The mice were obtained from Charles River Laboratories (Calco, Italy) and were maintained in laminar-air-flow rooms. Sterilised cages, bedding, food and acidified water were used for maintenance.

### Tumour line

IGROV-1 cells, from a human polymorphic and moderately differentiated ovarian carcinoma in an untreated patient, were kindly supplied by Dr J. Benard (Institut Gustave Roussy, Villejuif, France). In our laboratory, IGROV-1 was adapted to grow as ascites and maintained by i.p. injection of $2 \times 10^6$ cells/mouse in 0.4 ml of complete medium F12 (supplemented with 10% fetal calf serum and 5% antibiotics). Cells were collected from the donor mice using a heparinised syringe, suspended in sterile saline and centrifuged (1500 r.p.m. for 10 min). The supernatant was removed and the pellet resuspended in ammonium chloride solution (1:4 v/v) at 4°C for 10 min to lyse red blood cells. After washing twice in saline, cells were resuspended in complete medium and their number and viability were determined by trypan blue exclusion. Median survival time (MST) ranged from 17 to 25 days in different experiments.

### Drugs

Flavone acetic acid (FAA) kindly supplied by the National Cancer Institute, Division of Cancer Treatment (Bethesda, MD), was dissolved in 5% $NaHCO_3$ solution. Doxorubicin (DX) and cis-diamminedichloroplatinum (cisplatin, DDP), kindly supplied by Farmitalia-Carlo Erba (Nerviano, Italy), were dissolved in water and in saline, respectively. Drugs were delivered i.p. or i.v. at the volume of $10\,ml\,kg^{-1}$ of body weight. The optimal dose of FAA ($200\,mg\,kg^{-1}$) according to a schedule of 1 treatment every 4 days for a total of 3 times was used throughout the study (Giavazzi *et al.*, 1988). The two conventional cytotoxic drugs were administered according to schedules and at doses having proven efficacy against this tumour in our experience.

### Therapy studies

*Intraperitoneally growing tumour* Mice were injected i.p. with $2 \times 10^6$ cells in 0.4 ml of complete medium. Each experimental group consisted of 9–10 mice. Drug treatment was started 7 or 8 days after tumour cell inoculum. The percentage increase in median survival time (MST) in treated over control mice (T/C%) was used to assess drug effect.

*Subcutaneously growing tumour* Both flanks of mice were injected with $2 \times 10^6$ cells in 0.2 ml of complete medium. Between nine and 12 tumours were included in each group. Drug treatments started when tumours weighed more than 200 mg (day 10 or 14). Tumour weight (TW) was calculated according to Geran *et al.* (1972). The percentage tumour weight inhibition (TWI%) in treated mice was calculated 4 days after the last drug treatment according to the formula: 100-(mean TW in treated/mean TW in controls × 100).

*Experimental lung metastases* Mice were inoculated via the tail vein with $5 \times 10^4$ viable cells in 0.2 ml of complete medium. For counting of experimental metastases, mice were killed at established times (see Table III). After killing, a 15% solution of India ink in phosphate buffered saline (PBS) was injected into the bronchus of each mice. The removed lungs were bleached in Fekete's solution (Fekete, 1938) allowing the metastases to be counted easily as they formed discrete white nodules on the surface of the lung.

*Experimental liver metastases* Mice were inoculated in the spleen with $5 \times 10^5$ cells in 1 ml of medium according to Lafreniere and Rosenberg (1986). Briefly, mice were anaesthetised and their left flanks were prepared for surgery. A small, cutaneous incision was made and the spleen was carefully exposed and IGROV-1 cells injected under the spleen capsule via a 27-gauge needle. One minute after cell inoculation the spleen was removed and the abdominal cavity closed. At established times mice were given an injection in the tail vein of 0.5 ml of a 15% solution of India ink in PBS. The mice were then killed by cervical dislocation and their livers were harvested into vials containing Fekete's solution which bleached the liver, making the tumour deposits identificable as discrete white nodules against a black background of normal liver parenchyma.

Drug treatments started on day 3 or 10 in mice bearing lung and liver metastases. Drug efficacy was assessed by comparing the median numbers of colonies in treated and in control mice. Mice without colonies were excluded from calculation if present in both the control and treated groups because the absence of the liver colonies might be the result of failed inoculation. They were however included if present only in the treated groups because they may then be reasonably ascribed to drug efficacy. The number of mice per group is reported in the tables.

### Statistical comparison

Two tailed Student's *t*-test and Mann-Whitney Rank test were used for statistical analysis.

### In vitro *studies*

Cell survival was assessed by tetrazolium dye (MTT) assay (Alley *et al.*, 1988). In brief, cells were harvested from exponential-phase maintenance cultures, dispensed into 96-well culture plates (Costar Plastics 3799) in 100 µl volumes using a repeating pipette (Multipette 4780, Eppendorf) and treated with 10 µl of drug solution or medium for control wells. Each plate had eight control wells and eight wells for each dose. After incubation of the microtiter plates for 96 h, 10 µl of MTT working solution (5 mg $ml^{-1}$) was added to each culture well and cultures were incubated at 37°C for 4 h. The culture medium was removed from the wells and replaced with 100 µl of DMSO, using a multichannel pipette. The absorbance of each well was measured with a microculture plate reader (SLT Labinstruments, Austria) at 550 nm interfaced with an Apple computer. Preliminary experiments were performed to determine the appropriate seeding number of the cell line ($2.5 \times 10^3$ cells/well), after confirming the linear relationship between the absorbance and number of cells in the growth curve. The $ID_{50}$ was defined as the concentration of drug that produced 50% reduction of absorbance.

### Results

The effects of FAA treatment on IGROV-1 tumour xenografted i.p. or s.c. are presented in Table I. FAA was clearly inactive against the ascitic tumour, but inhibited the growth of established subcutaneous tumours.

The effects of FAA on experimental metastases of IGROV-1 tumour in lungs or in liver are reported in Table II. It can be seen that the drug is inactive against lung metastases when delivered either early (day 3) or late (day 10, at which time histological evidence of tumour nodules in the lungs was observed). On the contrary, FAA clearly reduced the number of liver colonies ($P < 0.01$ compared to the control mice) even when the drug treatments started at day 10.

The sensitivity to two conventional cytotoxic drugs, i.e. doxorubicin (DX) and cisplatin (DDP), of IGROV-1 tumour growing in various sites, is reported in Table III. IGROV-1 tumours respond to the two drugs in all the sites even though at different levels.

The results of the *in vitro* studies (Figure 1) clearly showed FAA to be less cytotoxic than DX against IGROV-1 cells, the $ID_{50}$ values being 20 and 0.05 µg $ml^{-1}$, respectively.

### Discussion

The results obtained in the treatment of the human IGROV-1 tumour clearly showed a site-dependent sensitivity to FAA. Significant antitumour effects occurred for tumours growing s.c. and in liver whereas i.p.- and lung-growing tumours failed to respond. In contrast to the inactivity of FAA, IGROV-1 cells growing i.p. did respond to locoregional treatment with two conventional cytotoxic drugs, thus confirming the marginal contribution of a direct cytotoxicity in the mechanism of action of FAA. These *in vivo* results are consistent with the observation that *in vitro* very high concentrations of FAA are required for cytotoxic effects on these tumour cells.

For s.c. growing murine tumours, the sensitivity has been possibly ascribed to a Tumour Necrosis Factor (TNF)-like action of FAA (Smith *et al.*, 1987; Finlay *et al.*, 1988), and recently we have demonstrated a critical role of TNF in FAA activity against murine colon tumour # 26 (Pratesi *et al.*, 1990). However, the possibility that FAA antitumour efficacy on this human carcinoma is mediated through TNF seems unlikely, since haemorrhagic necrosis was not visible in the tumours 24 h after FAA treatment. Moreover, T cells represent an important component of FAA efficacy (Pratesi *et al.*, 1990) and this mechanism may not be operating in nude mice, genetically lacking of T cells. Therefore, the basis of the activity of the drug against this human tumour xenografted s.c. in nude mice is unclear, but may be due to an effect on tumour blood flow as described in other tumour models (Evelhoch *et al.*, 1988; Bibby *et al.*, 1989*a*; Zwi *et al.*, 1989).

The most striking finding of this study was the dramatic difference in the efficacy of FAA against tumour cells growing in lungs or in the liver, whereas DX and DDP were

**Table I** Chemosensitivity of IGROV-1 human tumour to FAA (200 mg $kg^{-1}$)

| Tumor site | No. of mice (tumours) | Treatment Route | Treatment Days | %T/C[a] | %TWI[b] | P< |
|---|---|---|---|---|---|---|
| i.p. | 10 | i.p. | 7,11,15 | 97 | | 0.2 |
| s.c. | (11) | i.v. | 10,14,18 | | 70 | 0.05 |
| | (7) | | 14,18,22 | | 65 | 0.05 |

[a]% Increase of median survival time (MST) in treated (T) mice over control (C) mice. MST in controls was 20 days. [b]% Tumour weight inhibition in treated mice compared to control mice, measured 4 days after the last treatment.

**Table II** FAA (200 mg/kg, i.v.) activity against experimental metastases of IGROV-1 human tumour

| *Metastases in* | *Days of treatment* | *Day of evaluation* | *No. of mice with metastases/total* | *Median no.[a] of metastases* | *P<* |
|---|---|---|---|---|---|
| Lung | – | 28 | 7/8 | 31 (12–147) | |
| | 3,7,11 | 28 | 7/9 | 26 (1–216) | 0.1 |
| | – | 35 | 6/6 | 172.5 (29–250) | |
| | 10,14,18 | 35 | 7/7 | 188 (2–250) | 0.2 |
| Liver | – | 24–28[b] | 12/12 | 200 (2–250) | |
| | 10,14,18 | 24–28 | 8/11 | 29 (0–250) | 0.01 |

[a]Only numbers of metastatic deposits < 250 could be reliably counted; organs with deposit numbers > 250 were assigned an empirical number of 250. Ranges of values are given in parentheses. [b]Data pooled from two experiments.

**Table III** Sensitivity of IGROV-1 human tumour to standard cytotoxic drugs

| *Tumour site* | *Drug* | *Dose mg kg⁻¹* | *Treatment Route* | *Treatment Days* | *Response* | *P<* |
|---|---|---|---|---|---|---|
| i.p. | DX | 7.5 | i.p. | 7,14,21 | 208[a] | 0.01 |
| s.c. | DX | 5 | i.v. | 8,12,14 | 39[b] | 0.1 |
| lung | DX | 5 | i.v. | 3,7,11 | 0[c] (0–62) | 0.1 |
| liver | DX | 5 | i.v. | 10,14,18 | 12[d] (0–81) | 0.2 |
| i.p. | DDP | 6 | i.p. | 7,14,21 | 219[a] | 0.01 |
| s.c. | DDP | 4 | i.v. | 14,18,22 | 65[b] | 0.05 |
| lung | DDP | 4 | i.v. | 3,7,11 | 0[c] (0–6) | 0.05 |

[a]% Increase of median survival time (MST) in treated (T) mice over control (C) mice. MST in C was 20 days. [b]% Tumour weight inhibition in treated mice compared to control mice, 4 days after the last treatment. [c]Median number of metastases in lungs. Ranges of values are given in parentheses. Controls: 59 (7–94). [d]Median number of metastases in liver. Ranges of values are given in parentheses. Controls: 250 (2–250).

**Figure 1** IGROV-1 cells were exposed to drugs for 96 h. Cytotoxicity was evaluated by the tetrazolium derivative reduction (MTT) assay. DX: ●; FAA: ○.

active at both sites. The reasons for this difference remain unclear. Even though a cytotoxic effect of FAA *per se* seems unimportant in its antitumour efficacy, the high response achieved against liver colonies could be the result of metabolic activation of the drug to more cytotoxic compounds as observed *in vitro* (Chabot *et al*., 1989). Differences in metabolism between mouse and man (Cummings *et al*., 1989) must be considered, and may explain the lack of activity in 17 patients with liver metastasis (Kerr *et al*., 1989). As an alternative explanation of the different effect of FAA against liver and lung metastases, one might speculate that a critical threshold drug level at the tumour site is required for antitumour action. In fact, a higher peak level and area under the curve values (Damia *et al*., 1988), as well as a higher increase in NK activity (Wiltrout *et al*., 1988) in liver than in lungs of mice have been described.

In conclusion, in contrast to the effects of two conventional cytotoxic drugs, which were effective against IGROV-1 ovarian tumour in all the examined sites, the activity of FAA was critically dependent on the site of tumour growth indicating that mechanisms other than a direct cytotoxicity are determinants of FAA activity even against human xenografts.

The authors wish to thank Mrs L. Zanesi for typing the manuscript. This work was partially supported by a grant from Italian Research National Council (CNR) – PFO # 88.00826.44.

### References

ALLEY, M.C., SCUDIERO, D.A., MONKS, A. & 7 others (1988). Feasibility of drug screening with panels of human tumor cell lines using a microculture tetrazolium assay. *Cancer Res.*, **48**, 589.

BIBBY, M.C., DOUBLE, J.A., PHILLIPS, R.M. & LOADMAN, P.M. (1987). Factors involved in the anti-cancer activity of the investigational agents LM985 (flavone acetic acid ester) and LM975 (flavone acetic acid). *Br. J. Cancer*, **55**, 159.

BIBBY, M.C., DOUBLE, J.A., LOADMAN, P.M. & DUKE, C.V. (1989*a*). Reduction of tumor blood flow by flavone acetic acid: a possible component of therapy. *J. Natl Cancer Inst.*, **81**, 216.

BIBBY, M.C., PHILLIPS, R.M. & DOUBLE, J.A. (1989*b*). Influence of site on the chemosensitivity of transplantable murine colon tumours to flavone acetic acid (LM975, NSC 347512). *Cancer Chemother. Pharmacol.*, **24**, 87.

CHABOT, G.G., BISSERY, M.C. & GOUYETTE, A. (1989). Flavone acetic acid (LM-975; NSC-347512) activation to cytotoxic species in vivo and in vitro. *Cancer Chemother. Pharmacol.*, **24**, 273.

CHING, L.M. & BAGULEY, B.C. (1987). Induction of natural killer cell activity by the antitumour compound flavone acetic acid (NSC 347 512). *Eur. J. Cancer Clin. Oncol.*, **23**, 1047.

CORBETT, T.H., BISSERY, M.C., WOZNIAK, A. & 5 others (1986). Activity of flavone acetic acid (NSC-347512) against solid tumors of mice. *Investigational New Drugs*, **4**, 207.

CUMMINGS, J., DOUBLE, J.A., BIBBY, M.C. & 5 others (1989). Characterization of the major metabolites of flavone acetic acid and comparison of their disposition in humans and mice. *Cancer Res.*, **49**, 3587.

DAMIA, G., ZANETTE, M.L., ROSSI, C., MANDELLI, R., FERRARI, A. & D'INCALCI, M. (1988). Dose-dependent pharmacokinetics of flavone acetic acid in mice. *Cancer Chemother. Pharmacol.*, **22**, 47.

EVELHOCH, J.L., BISSERY, M.C., CHABOT, G.G. & 4 others (1988). Flavone acetic acid (NSC 347512)-induced modulation of murine tumor physiology monitored by in vivo nuclear magnetic resonance spectroscopy. *Cancer Res.*, **48**, 4749.

FEKETE, E. (1938). A comparative morphological study of the mammary gland in a high and low tumor strain of mice. *Am. J. Pathol.*, **14**, 557.

FINLAY, G.J., SMITH, G.P., FRAY, L.M. & BAGULEY, B.C. (1988). Effect of flavone acetic acid on Lewis lung carcinoma: evidence for an indirect effect. *J. Natl Cancer Inst.*, **80**, 241.

GERAN, R.I., GREENBERG, N.H., MACDONALD, M.M., SCHUMACHER, A.M. & ABBOTT, B.J. (1972). Protocols for screening chemical agents and natural products against animal tumors and other biological systems. *Cancer Chemother. Rep.*, **3**, 1.

GIAVAZZI, R., GAROFALO, A., DAMIA, G., GARATTINI, S. & D'INCALCI, M. (1988). Response to flavone acetic acid (NSC 347512) of primary and metastatic human colorectal carcinoma xenografts. *Br. J. Cancer*, **57**, 277.

HORNUNG, R.L., YOUNG, H.A., URBA, W.J. & WILTROUT, R.H. (1988). Immunomodulation of natural killer cell activity by flavone acetic acid: occurrence via induction of interferon α/β. *J. Natl Cancer Inst.*, **80**, 1226.

KAYE, S.B., CLAVEL, M., DODION, P. & 5 others (1988). Phase II trials of flavone-acetic acid (FAA) in patients with cancers of the breast, colon, lung, head and neck and melanoma. *Proceedings of ASCO*, **7**, 67.

KERR, D.J., KAYE, S.B., CASSIDY, J. & 8 others (1987). Phase I and pharmacokinetic study of flavone acetic acid. *Cancer Res.*, **47**, 6776.

KERR, D.J., MAUGHAN, T., NEWLANDS, E. & 4 others (1989). Phase II trials of flavone acetic acid in advanced malignant melanoma and colorectal carcinoma. *Br. J. Cancer*, **60**, 104.

LAFRENIERE, R. & ROSENBERG, S.A. (1986). A novel approach to the generation and identification of experimental hepatic metastases in a murine model. *J. Natl Cancer Inst.*, **76**, 309.

MANZOTTI, C. & PRATESI, G. (1988). Non-selective metastatic spread of a human ovarian adenocarcinoma xenografted in nude mice. Metastasis Congress, Heidelberg. Abstract no. 16A.

PLOWMAN, J., NARAYANAN, V.L., DONALD, D. & 4 others (1986). Flavone acetic acid: a novel agent with preclinical antitumor activity against colon adenocarcinoma 38 in mice. *Cancer Treat. Rep.*, **70**, 631.

PRATESI, G., MANZOTTI, C., DAMIA, G. & D'INCALCI, M. (1988). Response of chemically induced primary colon tumours of the mouse to flavone acetic acid (NSC 347 512). *Br. J. Cancer*, **58**, 144.

PRATESI, G., RODOLFO, M., ROVETTA, G. & PARMIANI, G. (1990). Role of T cells and tumour necrosis factor in antitumor activity and cytotoxicity of flavone acetic acid. *Europ. J. Cancer* (in press).

SMITH, G.P., CALVELEY, S.B., SMITH, M.J. & BAGULEY, B.C. (1987). Flavone acetic acid (NSC 347512) induces haemorrhagic necrosis of mouse colon 26 and 38 tumours. *Eur. J. Cancer Clin. Oncol.*, **23**, 1209.

WILTROUT, R.H., BOYD, M.R., BACK, T.C., SALUP, R.R., ARTHUR, J.A. & HORNUNG, R.L. (1988). Flavone-8-acetic acid augments systemic natural killer cell activity and synergizes with IL-2 for treatment of murine renal cancer. *J. Immunol.*, **140**, 3261.

WILTROUT, R.H. & HORNUNG, R.L. (1988). Natural products as antitumor agents: direct versus indirect mechanisms of activity of flavonoids. *J. Natl Cancer Inst.*, **80**, 21.

WORKMAN, P. & WARD, R. (1989). Tumour penetration by flavone acetic acid. Sixth NCI-EORTC Symposium, Amsterdam. Abstract no. 441.

ZAHARKO, D.S., GRIESHABER, C.K., PLOWMAN, J. & CRADOCK, J.C. (1986). Therapeutic and pharmacokinetic relationships of flavone acetic acid: an agent with activity against solid tumors. *Cancer Treat. Rep.*, **70**, 1415.

ZWI, L.J., BAGULEY, B.C., GAVIN, J.B. & WILSON, W.R. (1989). Blood flow failure as a major determinant in the antitumor action of flavone acetic acid. *J. Natl Cancer Inst.*, **81**, 1005.